# Comparable Instrumented Knee Joint Laxity and Patient-Reported Outcomes After ACL Repair: Response

**DOI:** 10.1177/03635465221144035

**Published:** 2023-03-01

**Authors:** Johannes Glasbrenner, Michael J. Raschke, Christoph Kittl, Elmar Herbst, Christian Peez, Thorben Briese, Philipp Michel, Mirco Herbort, Clemens Kösters, Benedikt Schliemann

**Authors’ Response:**

We appreciate the interest in our recent publication “Comparable Instrumented Knee Joint Laxity and Patient-Reported Outcomes After ACL Repair With Dynamic Intraligamentary Stabilization or ACL Reconstruction: 5-Year Results of a Randomized Controlled Trial”^7^ and the opportunity to respond to the letter to the editor by Runer et al.

The aim of the study was to compare anterior tibial translation (ATT), patient-reported outcome measures (PROMs), and complications/revision surgery after anterior cruciate ligament (ACL) repair with dynamic intraligamentary stabilization (DIS) in comparison with ACL reconstruction 5 years postoperatively. The study was designed and conducted as a single-center prospective randomized controlled trial. A total of 85 patients gave their consent to participate in the study, of which 83 patients (98%) were successfully followed for 2 years.^11^ After 5 years, however, 21 patients (25%) were lost to follow-up. Reasons for this were that patients moved to a different country (n = 2) or region (n = 5) or did not respond to the follow-up invitations (n = 14).^7^ The decrease in follow-up rate during the study period reflects the difficulty of completing a 5-year follow-up in a young and active study population, with frequent changes in residency for academic or professional reasons.^7^

Difference in ATT measured with the Lachman/Rolimeter test was selected during design of the study as a primary outcome measure because of its high sensitivity and diagnostic accuracy.^13^ Thus, this outcome parameter has been used for sample size calculation. As the follow-up rate at 5 years was lower than expected (n = 64; 75%) and given the exclusion of those with recurrent instability, only 46 patients were included in the final assessment of ATT and PROMs. Therefore, at the 5-year follow-up, the study might be underpowered.^7^

Regarding the title and conclusion of our publication, Runer et al’s letter to the editor raises the question whether ACL repair with DIS or ACL reconstruction would provide comparable results. ACL repair with DIS is the only modern technique of ACL repair that has been evaluated in a randomized controlled manner with a 5-year follow-up: in our recent publication, we found no statistically significant difference between ACL repair with DIS and ACL reconstruction regarding ATT and PROMs at 2 years.^7^ These results are in line with findings of a recent randomized controlled trial by Hoogeslag et al.^10^ Furthermore, these results are in accordance with the favorable findings regarding ATT and PROMs in various cohort studies with >2000 patients, as included in a systematic review by Ahmad et al.^1^

Biologically augmented ACL repair was compared with ACL reconstruction in a prospective randomized trial with a 2-year follow-up by Murray et al^14^: ATT and the International Knee Documentation Committee subjective score were comparable between groups, whereas a significantly higher mean hamstring muscle strength was found after ACL repair as compared with ACL reconstruction.

A major concern of historical techniques of ACL repair was the rate of recurrent instability.^4,5^ Although no significant difference between a modern technique of ACL repair and ACL reconstruction regarding recurrent instability was found in our recent publication nor in the randomized controlled trials of Hoogeslag et al^10^ and Murray et al,^14^ the concern of high rates of recurrent instability following ACL repair remains.^7^ However, evidence exists that careful patient selection may significantly reduce the rate of recurrent instability following ACL repair with DIS.^8,9,12^ Young age and a high activity level were identified as independent risk factors for failure of ACL repair in the studies by Krismer et al^12^ and Henle et al.^9^ Furthermore, statuses of the synovial sheet and tear localization were determined as prognostically relevant factors by Ateschrang et al.^3^ Henle et al found a recurrence rate of only 3.9% after ACL repair with DIS for proximal ACL tears in combination with a preinjury Tegner score <7. Such a failure rate is in line with current data regarding ACL reconstruction.^2,6^ However, it is well known that young age and high activity level are risk factors not only for treatment failures after ACL repair but also after ACL reconstruction.^2^ Thus, instead of focusing on the surgical technique only, surgeons should be aware of the importance of proper patient education, functional testing before clearing patients to return to sports, and other factors associated with ACL injuries or failures.

In conclusion, despite the increasing evidence regarding ACL repair with modern techniques, ACL reconstruction remains the gold standard in the treatment of ACL tears. However, based on the results of our recent publication and current literature, ACL repair with DIS is considered a feasible option to treat acute ACL tears, whereas the selection of the ideal patient for ACL repair should be investigated in further studies.

Once again, we thank Runer et al for their interest in our study and appreciate the scientific discussion on the important topic of ACL surgery and ACL repair.


Johannes Glasbrenner, MD
Michael J. Raschke, MD, Prof.
Christoph Kittl, MD
Elmar Herbst, MD, PhD
Christian Peez, MD
Thorben Briese, MD
Philipp Michel, MD
Mirco Herbort, MD, Prof.
Clemens Kösters, MD
Benedikt Schliemann, MD, Prof. *Department of Trauma, Hand and
Reconstructive Surgery, University Hospital Münster, Germany*

